# The Dawn of High-Throughput and Genome-Scale Kinetic Modeling: Recent Advances and Future Directions

**DOI:** 10.1021/acssynbio.4c00868

**Published:** 2025-04-22

**Authors:** Ilias Toumpe, Subham Choudhury, Vassily Hatzimanikatis, Ljubisa Miskovic

**Affiliations:** † Laboratory of Computational Systems Biology (LCSB), Ecole Polytechnique Fédérale de Lausanne (EPFL), Lausanne CH-1015, Switzerland

**Keywords:** Kinetic models of metabolism, Dynamical nonlinear systems, Kinetic rate laws, Generative machine learning, Synthetic biology, Systems biology

## Abstract

Researchers have invested much effort into developing kinetic models due to their ability to capture dynamic behaviors, transient states, and regulatory mechanisms of metabolism, providing a detailed and realistic representation of cellular processes. Historically, the requirements for detailed parametrization and significant computational resources created barriers to their development and adoption for high-throughput studies. However, recent advancements, including the integration of machine learning with mechanistic metabolic models, the development of novel kinetic parameter databases, and the use of tailor-made parametrization strategies, are reshaping the field of kinetic modeling. In this Review, we discuss these developments and offer future directions, highlighting the potential of these advances to drive progress in systems and synthetic biology, metabolic engineering, and medical research at an unprecedented scale and pace.

## Graphical abstract

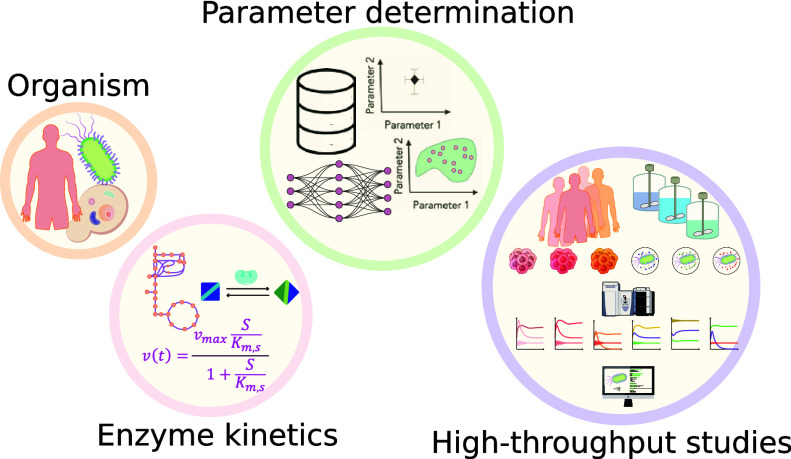

## Introduction

Metabolism plays a defining role in shaping the overall health of living organisms. Our ability to accurately describe cellular metabolism is important not only for biotechnological advances, such as the bioproduction of valuable compounds and environmental bioremediation, but also for developing new drugs, personalized therapies, and nutrition.^1,2^ The high complexity of metabolism combined with the impracticality of experimentally interrogating and quantifying all possible metabolic responses to genetic manipulations, mutations, and environmental conditions has spurred efforts in developing metabolic models that enable a systematic analysis of metabolism and offer insights into strategies for modifying cellular metabolism when necessary. Over the past 25 years, since the first genome-scale metabolic model (GEM) of *Haemophilus influenzae RD* was introduced,^3^ GEMs have become a cornerstone of systems-level metabolic studies.^4^ While these steady-state mathematical representations of metabolism have undoubtedly proven valuable in various domains of health and biotechnology,^1,5–8^ they lack crucial information on protein synthesis, enzyme abundance, and enzyme kinetics.^4,9^ As a result, they fall short of accurately predicting quantitative metabolic responses across many phenotypes,^10^ especially in the case of subtler gene modifications.^11^ Recent efforts have turned toward incorporating additional information in models, such as expression data and constraints^10,12–20^ as well as enzyme kinetics and regulation,^21–29^ to address these limitations.

The family of models accounting for the metabolic cost of transcription and translation, collectively referred to as Resource Allocation Models (RAMs),^30,31^ have significantly improved predictions under proteome limitations across various scenarios, including growth on different carbon sources,^14,32^ responses to stress conditions,^20^ and the metabolic burden of recombinant protein expression.^33^ RAMs leverage gene expression data to reliably predict the steady-state distribution of metabolites and fluxes. However, their reliance on steady-state assumptions and omission of enzyme kinetics makes them less adequate for capturing cellular responses under fluctuating conditions or transient states, where regulatory mechanisms—such as enzyme inhibition or activation, feedback loops, or changes in enzyme efficiency, play critical roles.

Kinetic models are particularly well-suited to describing intrinsically dynamic cellular processes that operate under continuously changing conditions.^2,34^ They are typically formulated as a deterministic system of ordinary differential equations (ODEs), depicting the balance between the production and consumption of metabolites within the network. In this way, kinetic metabolic models simultaneously link enzyme levels, metabolite concentrations, and metabolic fluxes. To validate and refine these models, their time-course and steady-state predictions can be compared to experimental data from various sources, including quantitative measurements of metabolite concentrations and metabolic fluxes over time for a single strain and physiological conditions or responses from multiple strains or conditions. This comparison is typically performed by assessing how well the model fits the data, its ability to generalize to unobserved responses, its robustness, and other relevant factors.

When developing kinetic models of metabolism, there are multiple modeling choices to consider, each affecting the model’s size and scope, predictive accuracy, and even interpretability. The dynamic changes in individual metabolite concentrations depend on the rates of the biochemical reactions in which they participate in. These reactions can be modeled as a sequence of elementary reaction steps, with each elementary step described through mass action kinetics.^35–37^ This approach allows for modeling enzymatic reactions with mechanistic details, including specific regulatory interactions and formation of enzyme–substrate complexes. However, it can become complex and computationally demanding when applied to reactions with multiple metabolites or large metabolic networks, as it requires defining many intermediate species, reaction parameters, and kinetic constants.^38^

Alternatively, one can model reactions with canonical and approximative rate laws that explain reactions without depicting intermediate species.^39–43^ Instead, these laws specify how the rate depends on the substrate and product concentration, enzyme activity, and regulatory effects. Although some mechanistic fidelity can be lost when using these rate laws, they require fewer kinetic parameters than when modeling reactions with elementary reaction steps. Parameters of such laws, such as Michaelis and inhibition constants, maximal velocities, and Hill coefficients, have clear and intuitive biochemical interpretations. Additionally, these laws can be applied to model a broad range of biochemical reactions, from enzyme–substrate interactions to cooperative binding and allosteric regulation. Allosteric regulation can also be modeled using dedicated frameworks designed to describe allostery.^44–46^

Ensuring thermodynamic consistency is another important aspect of kinetic modeling.^9,47^ The second thermodynamic law allows us to couple the directionality of reactions with metabolite concentrations directly because the reaction can operate only in the direction in which the difference in Gibbs free energy of the reaction is negative. The displacement of the reaction from the thermodynamic equilibrium dictates the reaction directionality, i.e., the ratio of forward and backward reaction rates.^38,48,49^ Since the thermodynamic properties of most reactions are not available, they are estimated using computational techniques such as the group contribution^50,51^ and component contribution^52,53^ methods.

The capability of kinetic models to capture how metabolic responses to diverse perturbations change over time enables studying dynamic regulatory effects on metabolism and complex interactions with other cellular processes. Equally important, these models enable direct integration and reconciliation of multiomics data, providing synergistic insights into metabolism and regulation. In contrast to steady-state models, which use inequality constraints to relate different omics data, kinetic models explicitly represent metabolic fluxes, metabolite concentrations, protein concentrations, and thermodynamic properties in the same system of ODEs, thus making the integration of these variables straightforward.^54^ For example, in steady-state models, inequality constraints derived from the second law of thermodynamics link metabolic fluxes with metabolite concentrations,^55^ whereas kinetic models directly couple them through rate equations. Similarly, given turnover number (*K*_*cat*_) values, the measured amounts of enzyme in the cell set upper bounds of metabolic fluxes in steady-state models. Kinetic models, in contrast, allow for direct incorporation of proteomics data by explicitly modeling enzyme kinetics.

Despite their recognized advantages and the growing interest in their development, the advancement and application of kinetic models have lagged behind those of steady-state models.^21,54,56–60^ This discrepancy stemmed primarily from the lack of available kinetic parameters and computational resources required to address this critical gap directly. However, rapid advancements are transforming this field, ushering in a new era where large kinetic models, including both large- and near-genome-scale models, will likely propel metabolic research forward.

## Metabolic Discovery with Kinetic Modeling

The substantial investment by the research community in kinetic modeling has been focused on advancing this field along three axes:

(i) Speed—recently proposed methodologies based on generative machine learning^54,60^ and novel nonlinear optimization formulations^29^ now enable the rapid construction of models and analysis of phenotypes, drastically reducing the time required to obtain metabolic responses and making high-throughput kinetic modeling a reality; indeed, current kinetic modeling methodologies achieve model construction speeds one to several orders of magnitude faster than their predecessors;
(ii) Accuracy—methodological advancements, the development of novel databases^61–65^ of enzyme properties and kinetic parameters, and greater access to high-performance computational resources have significantly improved the predictive capabilities of kinetic models, providing higher accuracy^66^ and enabling simulations that reliably mimic real-world experimental conditions;^28^
(iii) Scope—current modeling efforts are focused on developing large kinetic models that encompass a broad range of organisms and physiological conditions;^21,28,54,66^ creating genome-scale kinetic models is on the horizon, offering stronger performance by providing unique insights into metabolic processes and enabling the robust identification of optimal genetic and environmental interventions.

In the following, we examine these advancements more closely and explore their contributions to the progress of kinetic modeling and its potential applications.

### Recent Kinetic Modeling Methodologies

#### Classical Frameworks

Constructing kinetic models of metabolism is a multistage process in which each step presents unique challenges (Figure 1). Numerous methods and tools have been introduced that approach this workflow in diverse ways, reflecting the inherent complexity of metabolic systems (Table 1).

**Figure 1:**
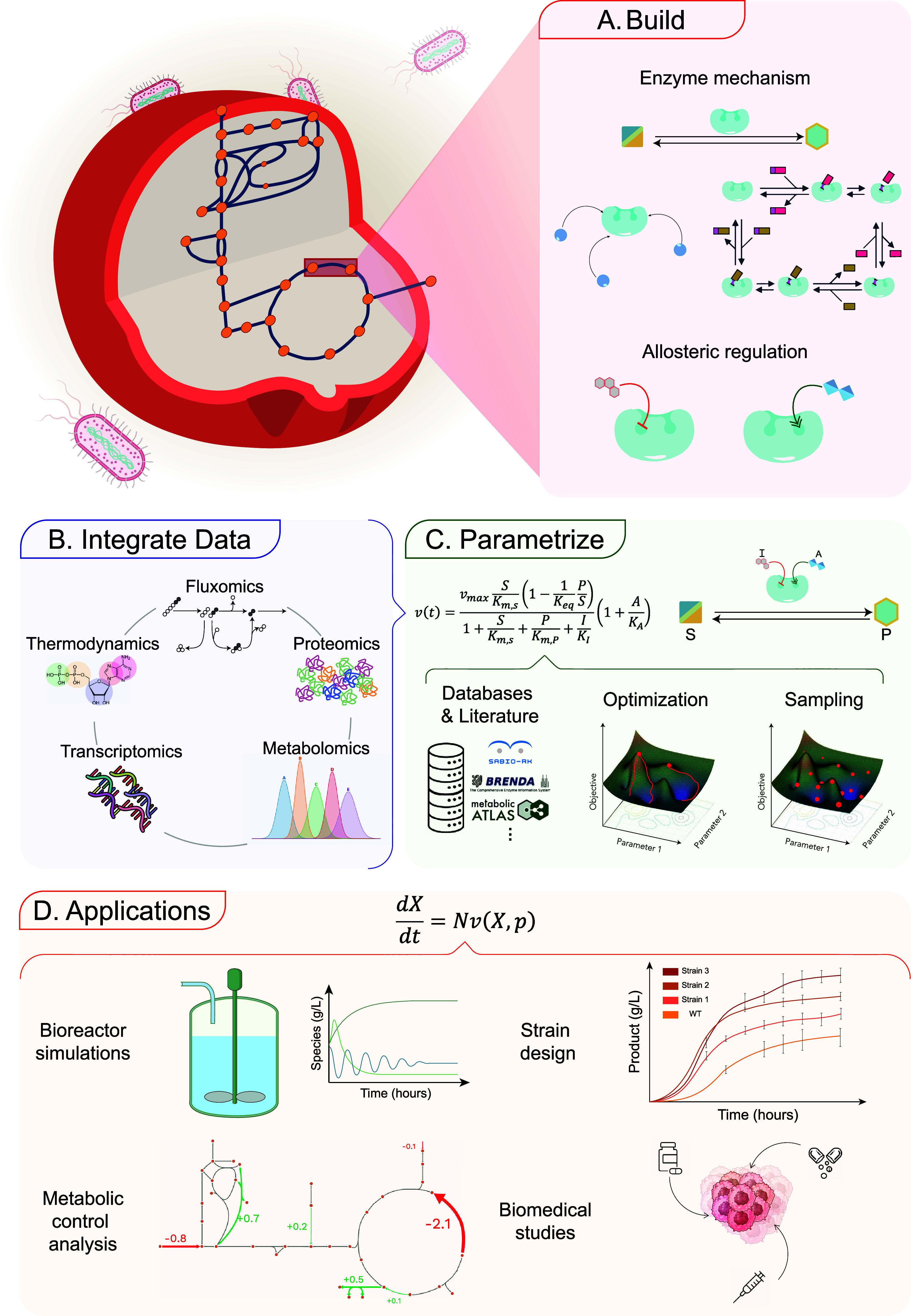
General framework for the kinetic modeling of metabolism. A. From a predefined network, each reaction flux is modeled based on enzyme mechanism expressions and known allosteric regulations. B. Integration of various types of omics data creates a physiology-specific kinetic model. C. Enzyme mechanisms are characterized by parameters that must be quantified. While some parameter information can be retrieved from databases and literature, additional estimation methods are necessary to parametrize kinetic models fully. D. A fully parametrized kinetic model, represented by a set of ordinary differential equations (ODEs), can be utilized for diverse applications across multiple fields and organisms.

**Table 1:** Comparative Analysis of Classical Kinetic Modeling Frameworks^a^

Method	Parameter determination	Requirements	Advantages	Limitations
SKiMpy^67^	Sampling	Steady state fluxes and concentrations; thermodynamic information	Uses stoichiometric network as scaffold; efficient; parallelizable; ensures physiologically relevant time scales; automatically assigns rate law mechanisms	Explicit time-resolved data fitting is not implemented
Tellurium^69^	Fitting	Time-resolved metabolomics	Integrates many tools and standardized model structures;	Limited parameter estimation capabilities
MASSpy^26^	Sampling	Steady state fluxes and concentrations	Well-integrated with constraint-based modeling tools; computationally efficient; parallelizable	Implemented only with mass action rate law
MASSef^71^	Fitting	In vitro/in vivo data for the kinetic parameters of the studied enzymes	Accurate approximation of the kinetic parameters	Not yet applied to large-scale models; computationally intensive; requires predefined rate law mechanisms
KETCHUP^29^	Fitting	Experimental steady state fluxes and concentrations from wild type and mutant strains	Efficient parametrization with good fitting; parallelizable and scalable	Requires extensive perturbation experiment data on the modeled physiology
Maud^72^	Bayesian statistical inference	Various omics data sets	Efficiently quantifies the uncertainty of parameter value predictions	Not yet applied to large-scale kinetic models; computationally intensive; requires predefined rate law mechanisms
Structural identification of kinetic parameters^73^	Fitting	Steady-state flux and concentration data	Analytically derives parameter values from a minimal set of experiments	Computationally intensive with increasing model size; prone to numerical issues; requires predefined rate law mechanisms.
pyPESTO^74^	Estimation with various techniques	Various experimental data; custom objective function for parameter estimation	Allows testing different parametrization techniques on the same kinetic model	Does not provide sensitivity and identifiability capabilities

a All of the methods are implemented in Python.

SKiMpy^67^ aims to make large kinetic models more accessible to the broader research community. This semiautomated workflow constructs and parametrizes models by using the network structure of stoichiometric models as a scaffold and assigning kinetic rate laws from a built-in library. It also allows for user-defined kinetic mechanisms. SKiMpy builds upon the ORACLE framework^68^ to sample kinetic parameter sets consistent with thermodynamic constraints and experimental data and prune them based on physiologically relevant time scales. Additionally, SKiMpy provides a robust tool for numerical integration across scales, from single-cell dynamics to bioreactor simulations.

Tellurium^69^ is a versatile kinetic modeling tool designed for applications in systems and synthetic biology. It supports various standardized model formulations and integrates external packages for the ODE simulation, parameter estimation, and visualization.

Another framework for kinetic model construction, MASSpy,^26^ uses, by default, mass-action rate laws but also allows users to define custom mechanisms for individual reactions. Built on COBRApy,^70^ MASSpy integrates the strengths of constraint-based metabolic modeling, enabling users to sample steady-state fluxes and metabolite concentrations effectively. The authors showcase the method’s practical capabilities through several case studies, demonstrating its utility in bridging constraint-based and kinetic modeling. MASSef^71^ also employs mass-action enzyme kinetics with a robust workflow that uses data fitting and optimization techniques to estimate sets of kinetic parameters. However, the reliance on mass action rate laws, which require many parameters, may lead to overfitting when the amount of data is small. The authors recommend using sampling methods to explore the parameter space effectively.

KETCHUP^29^ also uses an optimization-based parametrization that integrates stoichiometric information, enzyme mechanisms, and experimental data sets from different physiological conditions. The method constructs kinetic models by minimizing discrepancies between experimental and predicted flux distributions with a least-squares objective function. A novel feature of KETCHUP is its penalty on large kinetic parameter values, a strategy aimed at reducing overfitting and improving model accuracy. KETCHUP represents a significant advancement over its predecessor, K-FIT,^25^ since it decreased the computation time required by at least an order of magnitude while at the same time achieving better fitting. However, the method still depends on a large set of experimental data from different strains, which is not readily available for each physiology, especially for nonmodel organisms.

Maud^72^ follows a different path to estimate possible ranges for kinetic parameters by employing Bayesian statistical inference. It uses structural information and omics data sets and proposes ranges for kinetic parameter values, adding a layer of robustness to model predictions. This probabilistic approach effectively addresses inherent uncertainties in experimental data and allows for more comprehensive model exploration. However, further enhancements in computational efficiency are required to scale this method up to genome-scale kinetic modeling.

Another method^73^ for parameter estimation relies upon steady-state experimental data and closed-form algebraic expressions derived from the reaction rate laws, which are typically highly nonlinear. This approach determines both identifiable and nonidentifiable parameter sets and suggests a minimal number of experiments for the structural identification of all model parameters. However, the authors point out that current computational algebra techniques may struggle to analytically solve these expressions, which may limit the method’s scalability. To address this limitation, numerically based computational tools could be employed, enhancing the robustness of the workflow.

Applying and testing of various parameter estimation techniques on the same kinetic model can often be cumbersome. The tool pyPESTO^74^ addresses this challenge by providing a unified interface for a number of global and local optimizers. It supports the open-source interoperable format PEtab^75^ developed to standardize the process of building and parametrizing models in systems biology. pyPESTO accepts custom objective functions and utilizes various mathematical methodologies to quantify the uncertainty of the parameter values. The authors plan to further improve pyPESTO by adding a graphical user interface (GUI) to enhance user-friendliness and incorporating additional inference methods. This task is straightforward due to the tool’s modular design.

Although these new methods can generate large-scale networks, improve accuracy, and capture more complex biological phenomena, many challenges remain in the kinetic model building and parametrization workflow. To address some of these challenges, alternative approaches based on machine learning have been proposed.

#### Machine Learning Frameworks

In recent years, machine learning algorithms have been increasingly applied to analyze biological data, becoming a staple of systems and computational biology.^76–79^ Dynamical systems in biology are particularly amenable to machine learning because of their high-dimensional inputs and outputs, as well as their complex, coupled, and nonlinear behavior.^60^ Such methods have been successfully applied to approximate or accelerate different parts of the kinetic model building pipeline (Figure 2, Table 2).

**Figure 2:**
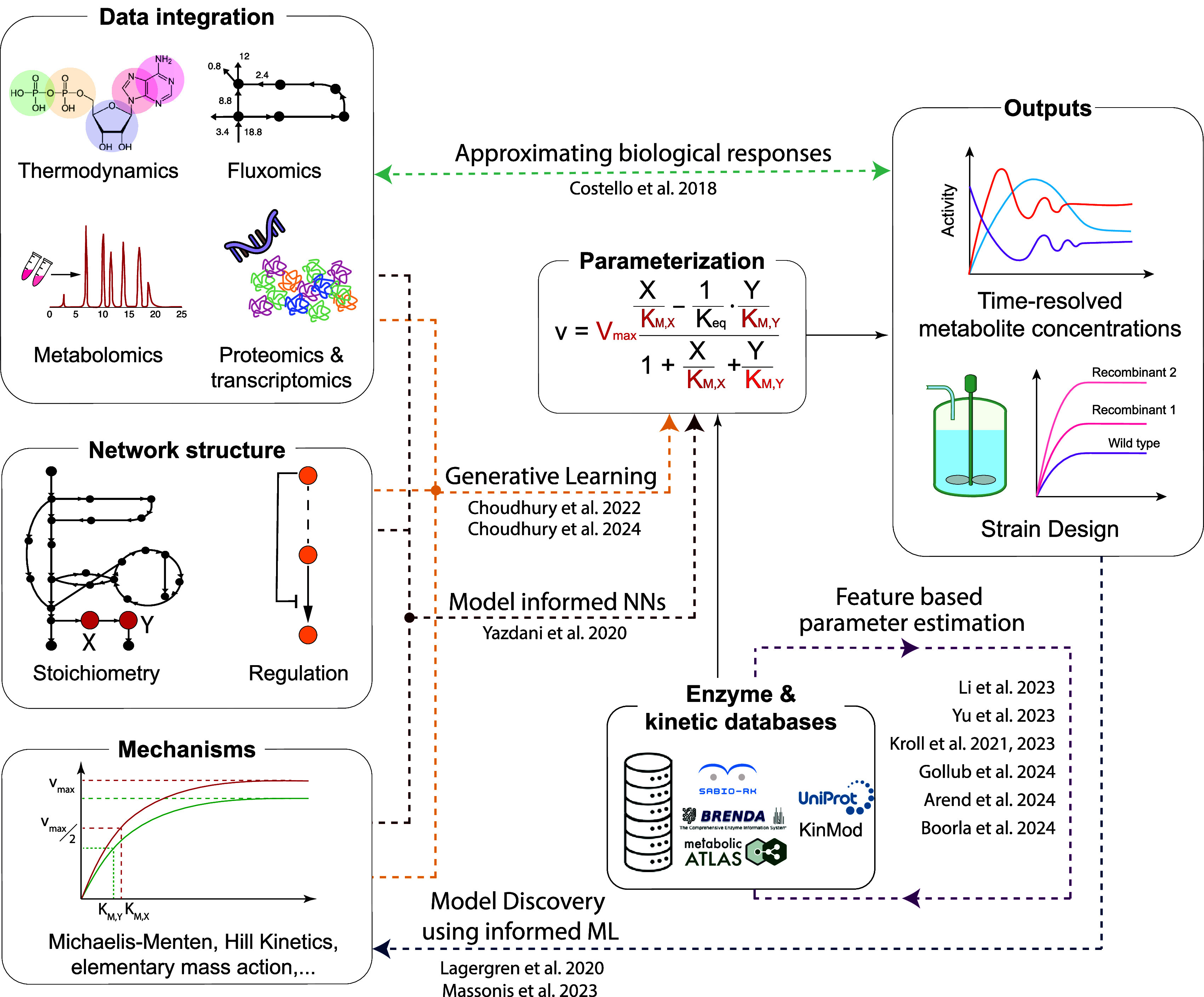
Applications of Machine Learning in the kinetic modeling of metabolism. Machine Learning has been successfully applied to approximate, accelerate, link, or consolidate (dashed lines) different steps of the classical kinetic model-building process (in boxes).

**Table 2:** Comparative Analysis of ML-Assisted Kinetic Modeling Frameworks

Class	Method (Application)	Requirements	Advantages	Limitations	Platform (hardware)
Deep Generative Learning	REKINDLE^60^ (parametrization)	Predefined model structure; steady state fluxes and concentrations; training data	Efficient, allows transfer learning across different physiologies, works on large scale models	Requires training data from other kinetic modeling frameworks; hyperparameter tuning required	Python (GPU)
	RENAISSANCE^54^ (parametrization)	Predefined model structure; steady state fluxes and concentrations	No training data required, efficient, easily integrates experimental kinetic data; parallelizable across CPU threads, works on large scale models; versatile	Hyperparameter tuning required for different metabolic models	Python (CPU)
Physics Informed Neural Networks (PINNs)	Yazdani et al.^86^ (parametrization) Lagergren et al.^89^ Massonis et al.^88^ (model discovery)	Model structure (parametrization); library of putative terms (model discovery); time-series data (training)	Works on limited data, Robust to noise and sparsity of data, can infer model terms	Requires a predefined library of candidate terms; effective for smaller models (<30 parameters)	Python, MATLAB, (CPU)
Feature-based parameter prediction	Li et al.^57^ Yu et al.^90^ Kroll et al.^91,92^ Gollub et al.^93^ Arend et al.^94^ Boorla et al.^95^ Sajeevan et al.^96^ (parameter estimation)	Uses kinetic parameters from databases like BRENDA, Sabio-RK, and UniProt as training data	Estimate parameters for different organisms and physiologies, model structure not required	Requires large training data sets; predictions lack validation through kinetic or constraint-based models.	Python, MATLAB, (GPU)
Approximating biological responses	Costello et al.^80^ (infer pathway dynamics from data)	Time resolved proteomics data	Bypasses the need for enzymatic mechanisms and stoichiometry	Limited mechanistic insight	Python (CPU)

The ability of machine learning algorithms to find and learn meaningful patterns from complex multidimensional data was recently used to bypass the need to have explicit enzyme mechanisms in kinetic models and directly predict the time evolution of metabolite concentrations from proteomics data for isopentenol- and limonene-producing *E. coli* strains.^80^

Machine learning has also been used in hybrid modeling workflows, combining mechanistic constraints and context-specific multiomics data. Generative learning-based workflows REKINDLE^60^ and RENAISSANCE^54^ provide a versatile and efficient way to generate kinetic parameters and integrate and reconcile omics and physicochemical data. These methods complement and improve upon traditional sampling-based frameworks by employing deep neural networks to perform stratified sampling of the kinetic parameter space and increase the incidence of models with the desired dynamic properties. These methods significantly improve sampling efficiency, surpassing traditional kinetic modeling techniques by several orders of magnitude. REKINDLE effectively parametrized different physiological phenotypes of *E. coli* central carbon metabolism using minimal training data through transfer learning,^81^ resulting in an even more significant decrease in sampling time compared to classical methods. RENAISSANCE, an evolutionary strategy^82^-based framework for parametrizing dynamic systems, improved upon REKINDLE by eliminating the need for training data from classical frameworks. RENAISSANCE was used to directly parametrize a large-scale kinetic model of the W3110 trpD9923 *E. coli* strain that satisfies the reference intracellular metabolic state and experimentally observed physiological constraints. It also allows for easy integration of experimental kinetic information from databases like BRENDA,^63^ consolidating them with missing or unknown kinetic parameters.

Another approach to modeling dynamic processes with machine learning involves incorporating information about these processes as constraints during optimization or training. Known as Physics Informed Neural Networks (PINNs),^83–85^ this approach includes equations that describe the underlying system and its derivatives as constraints in the optimization process. Including such physical laws or ‘priors’ in the optimization process acts as a strong form of regularization, preventing overfitting to sparse and noisy data. As a result, PINNs become more robust to noise and can produce reliable predictions even when trained on limited or incomplete data sets. Furthermore, incorporating these physical priors allows PINNs to infer values across the entire domain, leading to better generalization beyond the available data. A recent study used PINNs to parametrize a kinetic model of the yeast glycolysis pathway using noisy time-resolved concentrations for NADP and ATP only.^86^

Such ‘dynamically informed’ algorithms have also been applied for model discovery when the full structure of the biological system is not known *a priori.* ^87^ This is done by fitting the model’s output to a library of putative model structures while minimizing model complexity. A similar approach was used to rediscover the yeast glycolysis oscillator model,^88^ which consists of a system of 7 coupled ordinary differential equations (ODEs) with 29 parameters defining the kinetic properties of the system. These ODEs incorporate 23 distinct mathematical terms that represent linear and nonlinear interactions within the system. This method successfully recovered the structure of the oscillator kinetic model, accurately selecting the correct terms and the parameter values from a library of over 3000 candidate terms. The need for a predefined library of differential terms can be bypassed by integrating additional ‘parameter’ neural networks that approximate the functional terms of the model structure.^89^ This approach enables accurate simulation of diffusivities, growth, and delays in scratch assays—methods used to study cell culture motility in tissue regeneration or developmental biology studies, with various cell densities, providing insights into cell migration and interactions in culture.

### Databases and Parameter Estimation Methods

Publicly available databases like BRENDA,^63^ Sabio-RK,^61^ and UniProt^97^ represent extensive community efforts to measure, curate, and catalog biological data on enzymes, metabolites, and pathways. They provide information about enzyme sequences, Michaelis constants, *K*_*M*_, inhibition constants, *K*_*I*_, enzyme turnover numbers, *K*_*cat*_, and reaction types in the form of Enzyme Commission (EC) numbers. These resources are continuously updated and were recently complemented by novel databases like KinMod.^64^ KinMod catalogs omics data for over 9800 organisms, including associated enzymes (sequences, EC numbers), biochemical reactions, and compounds, totaling over 2 million data points. The enzymes are also annotated with regulator molecules and experimentally measured *K*_*I*_ values whenever available along with *K*_*M*_ values, thus providing a platform for comparing metabolic regulation among different organisms.

In addition to cataloging and providing access to enzymatic information, these databases also serve as vital resources for training data for estimating unknown kinetic and enzymatic parameters by using machine learning. Several such efforts have been undertaken recently by the community. Deep Learning-based methods like UniKP,^90^ CatPRED,^95^ and similar efforts^91,94^ use ‘features’ such as enzyme sequences, substrate and reactant structures, EC numbers, and protein structures (whenever available) to predict kinetic parameters such as *K*_*M*_, *K*_*I*_, *K*_*cat*_, and catalytic efficiencies, *K*_*cat*_/*K*_*M*_. ENKIE^93^ utilizes hierarchical Bayesian multilevel models to achieve similar predictions. Methods like DLKcat^57^ and TurNuP^92^ specialize in organism-independent predictions of enzyme turnover numbers using deep learning. RealKcat approaches enzyme kinetics prediction as a classification problem and uses optimized gradient-boosted decision trees to produce mutation-sensitive estimates of enzyme kinetics.^96^ The parameter predictions from DLKcat have been cataloged in the novel database GotEnzyme,^65^ which provides access to over 25 million predicted *K*_*cat*_ values across 8000 species.

These databases and estimation methods can complement each other in curating and developing new and existing databases of enzyme properties and kinetic information. Additionally, integrating the predictions from these methods with kinetic model-building frameworks will increase the model’s ability to capture experimentally observed physiologies and improve its predictions.

### Applications of Kinetic Modeling

Kinetic models encompass applications in predictive and explanatory contexts, which make them invaluable for metabolic engineering and biomedical research. Recent applications of kinetic metabolic models can be broadly categorized into two main areas: (i) prediction and design, which focus on fine-tuning cellular processes for optimal function and designing targeted modifications of organisms to achieve metabolic or therapeutic objectives, and (ii) biological discovery, which offers mechanistic insights into biological processes across various organisms.

#### Prediction and Design

A recent example of employing kinetic models to predict metabolic responses is the application of NOMAD to optimize anthranilate production in *E. coli*.^28^ This framework combines mixed-integer linear programming^98^ with nonlinear bioreactor simulations derived from large-scale kinetic models to design robust genetic interventions. It identified eight previously validated targets, accurately reproduced experimental observations on the reference and two recombinant strains, and proposed novel targets.

The NOMAD framework was also used to accelerate Design–Build–Test–Learn (DBLT) cycles in the design of *S. cerevisiae* strains for *p*-coumaric acid overproduction.^99^ As part of this process, nine kinetic models that incorporated omics data were developed and validated with batch reactor experiments. NOMAD proposed ten phenotypically robust strain designs with improved production capabilities. Out of these, eight strains demonstrated an increased yield of *p*-coumaric acid in experimental tests while maintaining comparable growth rates. This study showcased the capability of kinetic models in advancing metabolic engineering and synthetic biology.

*Clostridium thermocellum* is another organism of interest in industrial biotechnology, and one study used kinetic models of its core metabolism to identify bottlenecks in ethanol production.^100^ The models were built using flux profiles calculated from ^13^C metabolic flux analysis and the K-FIT^25^ algorithm to estimate possible sets of kinetic parameter values. Using these models, the authors iteratively examined whether different types of competitive inhibition better explained the experimental data. This approach identified regulatory constraints that were then validated through ensemble-docking algorithms. This study shows the capabilities of kinetic models to perform hypothesis testing and assess alternative biological mechanisms.

A large-scale kinetic model of *Pseudomonas putida* metabolism was developed and used in two studies to (i) successfully capture the experimentally observed metabolic responses to several single-gene knockouts of a wild-type strain of *P. putida* KT2440 growing on glucose and (ii) propose metabolic engineering interventions for improved robustness of this organism to the stress condition of increased ATP demand.^101^ Additionally, this work introduced a novel set of constraints within thermodynamics-based flux analysis that allow for considering metabolite concentrations in several compartments as separate entities.

Another study^102^ focused on the central carbon metabolism of *P. putida* KT2440 to identify targets for acetyl-CoA accumulation. They built an ensemble of kinetic models by integrating thermodynamic, fluxomic, and metabolomic information, as well as known allosteric regulations. To validate the prediction capabilities of the ensemble, they performed *in silico* metabolic knockouts and perturbations and compared the results with previous experiments. They then used Metabolic Control Analysis^103,104^ (MCA) to identify which reactions have the most effect on acetyl-CoA availability. The final design strategy proposed a dynamic downregulation of genes encoding enzymes citrate synthase (CS) and acetyl-CoA carboxylase (ACCOAC), which was experimentally implemented using CRISPRi, resulting in an 8-fold increase in acetyl-CoA concentration within the cells.

Kinetic models of mammalian cells have been used to identify enzyme adjustments that would reverse glucose flux in these cells.^105^ Additionally, kinetic models can aid human health by identifying metabolic vulnerabilities and potential targets to redirect metabolism toward a healthier state, offering valuable insights for addressing metabolic diseases. For example, kinetic modeling was applied to β-cell metabolism^106^ to identify metabolic fluxes impacting insulin secretion. This work highlighted potential metabolic drug targets and suggested future extensions that can model cellular heterogeneity and environmental interactions.

#### Biological Discovery

Kinetic models can inherently capture many aspects of biological complexity, making them an excellent tool for explaining biological phenomena at a systems level. This has naturally led to applications of kinetic models that test hypotheses and elucidate complex metabolic interactions. For example, in a series of studies,^107,108^ an ensemble of kinetic models for *Synechocystis sp. PCC 6803* was screened using multiple layers of omics data, identifying the enzyme phosphoglycerate kinase (PGK) as a target for ethanol production enhancement. Experimental overexpression of PGK confirmed these predictions, and the same models were used to postulate novel allosteric interactions, which were also experimentally validated.

A model of glycolytic dynamics in *Saccharomyces cerevisiae* was developed to explain the different responses of central carbon metabolism.^109^ An ensemble of models was parametrized using chemostat experiments and carbon flux measurements. The models could reproduce the behavior of a growing cell when compared to data measurements and suggested that the system is robust against parameter uncertainty. Any deviations from literature parameters were hypothesized to stem from missing regulatory mechanisms, particularly for pathways not included in the central carbon metabolism. Additionally, this work showcased the versatility of kinetic models by creating a metabolic-CFD hybrid model capable of estimating the dynamics of intracellular concentrations in a bioreactor setting. In another study, kinetic models were used to explain the metabolic differences between yeast strains.^66^ Specifically, two large kinetic models for distinct *Saccharomyces cerevisiae* strains were parametrized and validated since they could capture a significant portion of the fitted data set fluxes. The models revealed key metabolic differences between the strains, primarily in enzymes of the TCA cycle, glycolysis, and arginine and proline metabolism. In another study,^110^ a more compact kinetic model of *S. cerevisiae*’s lipid metabolism was manually built to describe all the necessary pathways for lipid homeostasis and overproduction. The model was parametrized by fitting lipidomic data of closely related strains and was used for hypothesis testing. This led to the identification of a futile cycle in the triacylglycerol biosynthesis pathway. The model also explained successful and unsuccessful strain designs explored in the literature.

To understand glycolytic flux control differences in healthy and cancer cells, this study^111^ constructed kinetic models describing glycolysis in healthy and cancer cells. While many controlling enzymes were shared, hexose-6-phosphate isomerase emerged as a critical driver of cancer glycolysis, suggesting its potential as a therapeutic target. Another study^34^ emphasized the limitations of constraint-based models in capturing human metabolic variation, proving the need for whole-cell kinetic models. Using patient metabolomics data, the authors created personalized erythrocyte metabolic models for 24 individuals, demonstrating how kinetic parameters could connect genotypic variations to metabolic phenotypes. This approach facilitated drug target simulations and offered insights into off-target drug effects.

A recent study of lactate homeostasis^112^ demonstrated the ability of kinetic models to investigate complex biological mechanisms. The authors developed a simple yet highly curated model, parametrized using experimental data from insulin pulses in mice. The model was used to assess the robustness of lactate homeostasis and study the effects of the removal of known regulatory mechanisms. While the model was robust to concentration and parameter perturbations, removing some inhibitory effects led to deregulated physiology, highlighting regulatory interactions that would be difficult to uncover through experiments alone.

In a different, more theoretical approach,^113^ MCA was performed on a simple model of upper glycolysis and a core model of *Escherichia coli* to demonstrate that the distribution of control over metabolic pathways cannot be explained with stoichiometry alone but requires information from enzyme kinetics and regulatory mechanisms. The study also demonstrates that large modifications in system parameters, such as enzyme activities, result in significant changes in the control distribution, which can be quantified only using approaches like MCA. The authors propose that a unified workflow incorporating predictions from GEMs and MCA in the future can assist in systematically identifying drug targets.

Kinetic modeling has also found an application in cell-free systems, where it has been used to optimize butanol production.^27^ A large-scale kinetic model developed in this context accurately predicted experimental outcomes and identified aldehyde/alcohol dehydrogenase as the primary bottleneck, guiding efforts to improve the production efficiency. Another intriguing application, however, takes an entirely different approach. Sabzevari et al. applied multiagent reinforcement learning (MARL) to tune enzyme abundance, optimizing growth in *E. coli* and enhancing L-tryptophan production in *S. cerevisiae*.^114^

Together, these applications highlight the capability of kinetic models to make accurate predictions and generate mechanistic insights. As the model-building workflows become more versatile and efficient, we expect a broader use of kinetic models for applications across different biological systems.

Readers are encouraged to consult elsewhere for a more detailed account of earlier research in this field.^115–117^

## Future Directions

The complexity of metabolism and its diverse physiological manifestations drive continuous advancements in kinetic modeling. Recent breakthroughs demonstrate how machine learning and data science transform the field, enabling more accurate parameter estimation and accelerated large kinetic model construction.^54,60,86^ Additionally, CPU and GPU performance advances now support computational methods previously deemed impractical, facilitating more comprehensive studies of metabolic dynamics.

Generative machine learning, optimization, and statistical approaches now permit the efficient construction of large nonlinear kinetic models of metabolism, positioning us on the brink of realizing genome-scale kinetic models in the near future.^29,54,60^ Kinetic models, much like or even more so than constraint-based models that incorporate kinetic parameters,^59^ are likely to unravel the genotype-phenotype relationship to a degree where we can design genotypes with targeted phenotypes. As models continue to increase in size and detail, challenges related to model calibration and characterization are expected to intensify, particularly due to limited omics data coverage. For example, metabolomics and proteomics currently detect and quantify only a fraction of metabolites and proteins.^118,119^ Additionally, as models expand to include larger metabolic networks and peripheral pathways, the number of uncharacterized kinetic parameters will likely grow due to the inclusion of reactions and interactions from poorly studied pathways for which corresponding kinetic parameters may not yet be available. Addressing these limitations will require the integration of diverse and extensive omics data sets alongside other complementary sources of information, such as extracellular medium composition, physicochemical properties, and expert knowledge, potentially aided by advanced data science techniques and new databases.

Beyond offering detailed mechanistic insights and quantitative predictions of metabolic phenomena, large kinetic models will allow us to explore the cross-talk of metabolism with dynamical systems such as transcription, translation, signal transduction, and cell division, which operate on vastly different time scales.^120,121^ It is challenging to model these processes and balance their different time scales without introducing unnecessary stiffness. Resolving this challenge is important for capturing the intricate interplay between these cellular processes, as stiffness can make solving these models and obtaining accurate solutions computationally demanding.

Along these lines, connecting large kinetic metabolic models with kinetic models of other cellular processes, especially RAM and signaling models, appears imminent given the current advancements in modeling techniques. Such an integration would address the critical need for collaboration across disciplines investigating metabolism, gene expression, signaling, and other cellular processes to deepen our understanding of cellular biology. For instance, combined kinetic models of metabolism and gene expression could facilitate exploring complex regulatory interactions, test hypotheses about putative feedback loops, and improve predictions of phenotypic responses to genetic and environmental changes such as productivity of target chemicals or stress adaptation. Similarly, integrated models of metabolism and signaling can play a crucial role in therapeutic applications by predicting how drugs might influence both metabolic and signaling pathways. This approach could lead to the identification of novel drug targets or therapeutic interventions for diseases involving dysregulated metabolism and signaling, such as cancer or metabolic disorders. Ultimately, all these efforts lead toward achieving comprehensive kinetic models of the whole cell, building upon existing steady-state counterparts.^122–124^ A key help in this pursuit will be the advancements in modeling the dynamic processes of metabolism, which can be readily applied to other cellular processes.^125^

The improved efficiency in constructing large kinetic models creates new opportunities to utilize the temporal evolution of phenotypic changes as an additional layer of constraints, reducing the uncertainty in experimentally uncharacterized omics data. A recent study highlights that dominant time constants of metabolic responses can enhance the characterization of unmeasured intracellular fluxes and metabolite concentrations.^54^ It is essential to note that metabolism and other cellular processes operate on a hierarchy of time scales,^126^ with metabolic pathways evolving at different rates. For example, rapid metabolic processes such as glycolysis and the electron transport chain are characterized by small time constants. In contrast, processes such as DNA, RNA, and protein synthesis occur at slower time scales. By integration of a broader range of such dynamic constraints, future research could more accurately narrow the allowable ranges of not-measured omics quantities. This approach also has the potential to guide experimental design, enabling targeted data collection to minimize uncertainty further and improve the predictive power of the kinetic models.

With their ability to predict time-resolved responses in systems where multiple processes act simultaneously across different time scales, kinetic models are particularly suited for studying multicellular systems and interactions among organisms. For instance, in host–pathogen interactions, the metabolic processes of microbes and parasites typically take place on time scales different from those of human cells. Integrated kinetic models of the human host cell with the embedded pathogen cell(s) will allow us to explore, postulate hypotheses, and provide learnings into how the pathogen interferes with the host metabolism and the host’s regulatory loops. Ultimately, these efforts may help identify potential strategies to enhance the host’s ability to combat pathogenic infections.

Kinetic models may also be instrumental in deciphering the mechanisms underlying the dynamics and functioning of multicellular systems and microbial communities. This area faces challenges originating from our incomplete understanding of the functional roles and interactions of the consortia species,^127^ as well as the sheer diversity and number of species involved. For example, the gut of a human individual harbors a few hundred distinct microbial species out of the thousands identified across the entire human population.^128^ To analyze such complex systems, it may be judicious to focus on small- or midscale kinetic models tailored to the systems of interest or to use metabolic reactions that summarize species interactions—a concept recently introduced in host–pathogen studies.^129^ Progressing in such efforts will probably require developing automated workflows for high-throughput data integration and the creation of strain- and context-specific kinetic models. Establishing such workflows will also be of great interest to personalized medicine applications, as it will allow modeling large patient cohorts and capturing the unique characteristics of each individual.

Given the success of initial applications of generative machine learning in kinetic modeling, these techniques are likely to represent a cornerstone for future advancements in this field. As the volume of generated biological data continuously grows, data science methods for analyzing such data sets will likely gain prominence. However, the suitability of the model structure will remain important depending on the specific objectives. Generative learning methods employed in kinetic modeling^54,60^ excel at parametrizing large kinetic models that consistently align with reference omics data. Nevertheless, they rely on predefined mechanistic constraints and network structures. Recently proposed mechanistically informed approaches can predict the mathematical structure of dynamic systems by incorporating a library of putative mechanisms into the optimization process.^88^ However, these methods are currently limited to smaller systems due to the rapid expansion of possible model configurations as the system complexity grows. To address these challenges, a synergy between generative learning and mechanistically informed approaches is essential. A conceptual ideal would involve fitting time-series omics data, e.g., metabolomics, through simultaneous kinetic structure discovery and parametrization, combining the strengths of both methodologies.

While neural networks (NNs) have proven effective in various kinetic modeling applications (Figure 2), their inherent black-box nature complicates interpreting their predictive mechanisms. One approach to overcome this limitation is integrating biological knowledge distilled from computational^130,131^ or experimental biological evidence into the architecture of these NNs. In previous studies, internal connections of sparse multilayer perceptrons and recursive NNs have been restricted based on biological data or prior knowledge, and such biologically informed NNs have been successfully used to model cell’s hierarchical structure and function,^132^ signaling,^133^ and predict cell state from single-cell RNA-seq data.^134^ This approach reduces the number of parameters to estimate, and, in some cases, provides a mechanistic understanding of the studied systems.^79^

Another approach to improving interpretability is equipping NNs with attention layers^135^ to gain further insights into their outputs. The self-attention mechanism in transformers allows for the identification of which factors most influence the kinetic parameters and the discovery of dependencies among these parameters. However, unlike kinetic models that involve continuous data, transformers are designed to treat discrete data such as protein sequences. Therefore, modifying attention layers or hybridizing with other ML models may be necessary to handle continuous data effectively.

Exploring the latent space of NNs,^136–138^ used in frameworks like RENAISSANCE, is yet another promising approach to improving interpretability. Analyzing this high-dimensional space allows us to understand better how NNs organize and process input data. Such exploration can help us uncover hidden patterns, such as higher-order relationships between kinetic parameters and gain mechanistic insights into the underlying biological processes.

Like any other field, machine learning for kinetic modeling requires ample, high-quality, and well-organized data, as the performance of the training process critically depends on the characteristics of the training data set. While data augmentation is valuable for addressing data scarcity and enriching training diversity—particularly for capturing out-of-equilibrium and out-of-distribution dynamics^139^—it should not replace experimental data outright. The quantity of data required is also system-specific and must be determined empirically, as the ‘learnability’ of different dynamical systems varies. The success of kinetic modeling, particularly the integration of machine learning in this field, relies heavily on strong collaboration between experimentalists and modelers. One of the biggest challenges in future kinetic modeling will likely be to align modeling efforts with experimental advancements, ensuring access to high-quality, curated, and consolidated data.

Unlike fields with well-established benchmarks and databases, such as ImageNet^140^ and MNIST^141^ in computer vision, kinetic modeling lacks universally accepted benchmarks, which hinders efforts to standardize and streamline progress in this field. Although databases of models^62,64^ and comparative studies^142,143^ have emerged in the literature, benchmarking kinetic models remains inherently challenging due to (i) variability in experimental conditions, measurement techniques, and studied organisms, which complicates standardized comparisons; (ii) difficulties associated with comparing models of different scales, studied metabolic subsystems (e.g., glycolysis, pentose-phosphate pathway), and levels of detail (mechanistic versus coarse-grained models); and (iii) the absence of standardized data sets. These challenges also affect machine learning applications, which rely on large, consistent data sets that are scarce in biochemical kinetics. Thus, coordinated efforts from the kinetic modeling community are required to develop standardized data sets and establish robust benchmarking protocols to overcome these challenges and advance this field.
